# AnalogExplorer2 – Stereochemistry sensitive graphical analysis of large analog series

**DOI:** 10.12688/f1000research.7146.1

**Published:** 2015-10-09

**Authors:** Ye Hu, Bijun Zhang, Martin Vogt, Jürgen Bajorath

**Affiliations:** 1 Department of Life Science Informatics, B-IT, LIMES Program Unit Chemical Biology and Medicinal Chemistry, Rheinische Friedrich-Wilhelms-Universität, Bonn, Germany

**Keywords:** Medicinal chemistry, analog series, computational design, graphical analysis, structure-activity relationships, open access software

## Abstract

AnalogExplorer is a computational methodology for the extraction and organization of series of structural analogs from compound data sets and their graphical analysis. The method is suitable for the analysis of large analog series originating from lead optimization programs. Herein we report AnalogExplorer2 designed to explicitly take stereochemical information during graphical analysis into account and describe a freely available deposition of the original AnalogExplorer program, AnalogExplorer2, and exemplary compound sets to illustrate their use.

## Introduction

In medicinal chemistry, analog series are typically analyzed in R-group tables. Once analog series become so large that they are difficult to represent and study in conventional R-group tables, computational tools are indispensable for their exploration. Therefore, different computational methods have been introduced for graphical analysis of analog series ^1– 9^. Many of these approaches are based on the determination of maximum common substructures (MCS) of compound series and focus on substituents of common cores, while others employ the matched molecular pair ^10^ formalism to define analog series. Among these computational methods is AnalogExplorer ^9^, which has been designed to systematically organize and graphically analyze analog series and associated structure-activity relationship (SAR) information. AnalogExplorer initially identifies analog series on the basis of hierarchical molecular scaffolds ^11^ and then determines their MCS for further analysis. Accordingly, the method is not limited to the study of individual analog series but can also be applied to extract series from structurally heterogeneous compound sets. For example, AnalogExplorer is directly applicable to late-stage lead optimization sets that often contain multiple series with large numbers of analogs.

Herein we introduce AnalogExplorer2, an extension of the approach, which explicitly considers stereoisomers during graphical analysis, providing a detailed account of stereochemistry and its influence on SARs. AnalogExplorer2 is publicly available. Hence, we also report an open access deposition of the original AnalogExplorer program and AnalogExplorer2 as well as exemplary data sets assembled to illustrate the workflow of graphical analysis and help users become familiar with the program ^12^.

## Methodology

### Organization of analog series

**Figure 1.**
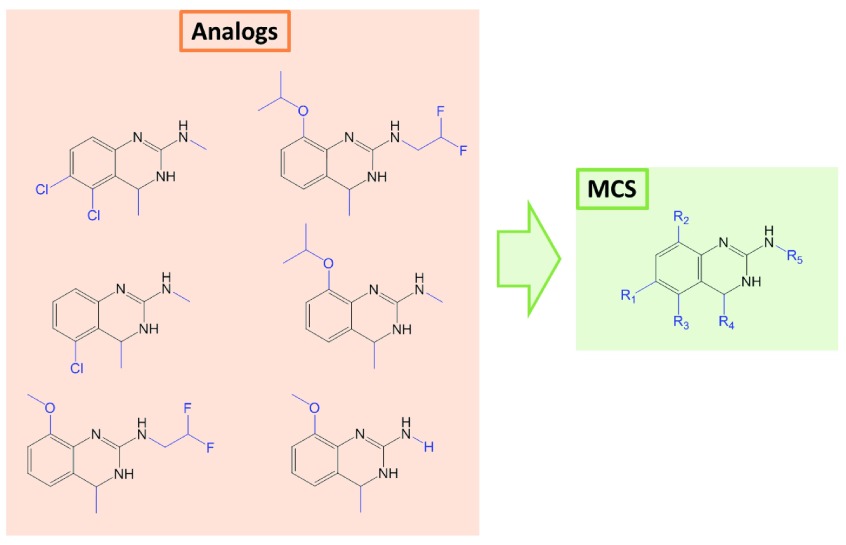
Maximum common substructure (MCS). Shown are six structurally analogous compounds represented by their MCS (right) with five substitution sites (R1–R5). For each compound, the corresponding substituents are highlighted in blue.

AnalogExplorer systematically determines substitution sites or site combinations in analog series and divides series into subsets having varying R-groups at the same site(s). Analog series are initially identified on the basis of hierarchical molecular scaffolds ^11^. For a series of analogs sharing the same scaffold, the maximum common substructure (MCS) is then determined, as illustrated in Figure 1, and used for R-group decomposition in order to index and identify all substitution sites and the respective R-groups for each compound in a series.

On the basis of the MCS, an analog series is divided into subsets of compounds with varying R-groups at the same substitution site or site combination. The organization is compound-based such that each member of a series only occurs in one subset. Unique compound subsets provide the basis for graphical analysis, as discussed in the following. Further methodological details are provided in the original AnalogExplorer reference ^9^.

### Graphical components

**Figure 2.**
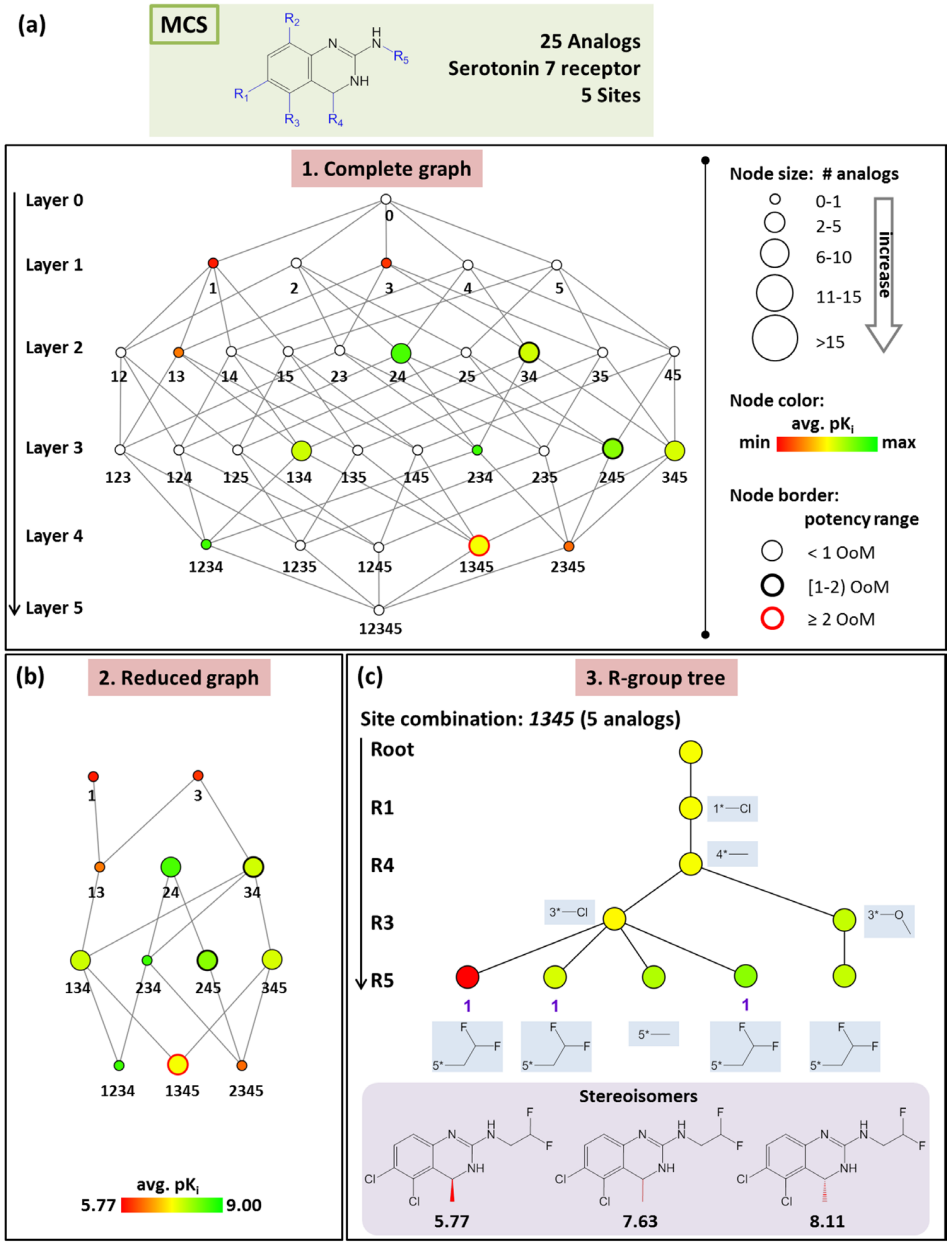
AnalogExplorer graphs. ( **a**) Shown is the *complete graph* for a series of 25 analogs active against serotonin 7 receptor. ( **b**) The *reduced graph* is displayed obtained from ( **a**) by removing all empty nodes and edges between them. ( **c**) The *R-group tree* for substitution site combination *1345* is shown. All R-groups are provided for individual tree nodes. Stereoisomers and their corresponding pK _i_ values are given at the bottom. Abbreviation: OoM, order of magnitude.

 AnalogExplorer consists of three graphical components. The *complete graph* ( Figure 2a) captures all possible substitution sites and site combinations for a series following R-group decomposition (by design it is a directed acyclic graph). Each node represents a substitution site or site combination and all compounds with varying R-groups at the site(s). The root node *0* corresponds to a (hypothetical) compound with no R-group at any site. Node *1* represents analogs that only contain R-groups at R1 and node *12* compounds with R-groups at R1 and R2 etc. Nodes are arranged in different layers. For example, layer 1 consists of all nodes with one substitution site and layer 2 of all nodes with two sites. Edges between nodes in adjacent layers indicate all possible subset relationships, i.e. an edge is drawn if the substitution site(s) represented by a node is a subset of a site combination of another node. As indicated in Figure 2a, nodes are scaled in size according to the number of analogs comprising the subset they represent and color-coded according to the mean potency of the analogs. In addition, node border thickness indicates the potency range covered by a subset. Furthermore, white (empty) nodes correspond to possible site combinations for which no analogs are currently available within a given series. In the *reduced graph*, all empty nodes and connecting edges are removed for clarity ( Figure 2b). Thus, the reduced graph provides a convenient format for the analysis of individual series. As the third graphical component, *R-group trees* are provided for each substitution site and site combination, as illustrated in Figure 2c. In the R-group tree, substitution sites for a given subset are arranged in different layers, the order of which is determined by the number of unique R-groups at each site. All R-groups are displayed in the tree. Each leaf node represents an analog (colored according to its potency). Intermediate nodes represent subsets of analogs sharing the same substituents at corresponding site(s) (and are colored by mean analog potency).

Given its design, AnalogExplorer provides a systematic hierarchical organization of all possible substitution sites or site combinations for an analog series (complete graph) and enables the elucidation of SAR patterns within the hierarchy (reduced graph) and at further increased resolution for analog subsets (R-group trees). The approach is particularly suitable for the analysis of large analog series because subsets of such series associated with interesting SAR information can be selectively displayed and analyzed.

### Stereochemical information

The explicit consideration of stereochemistry during graphical analysis at the level of R-group trees is the major methodological enhancement of AnalogExplorer2 (in addition to further increased consistency of compound mapping to MCS considering intra-molecular symmetry). In the original R-group tree structure, nodes located in the same layer and originated from the same parent node are associated with distinct R-groups. Therefore, stereoisomers having the same substituents are combined into a single leaf node. Hence if a terminal node is associated with more than one compound, stereoisomers are present. In AnalogExplorer2, stereoisomers are explicitly considered, as illustrated in Figure 2c. Each stereoisomer is represented by a single node and stereoisomers belonging to the same subset (i.e. compounds with different stereochemistry at the same site) are identified by a unique index (i.e. ‘1’ for the three stereoisomers in Figure 2c). If different subsets of stereoisomers are present in an R-group tree, incremental indices are used to identify and distinguish them (i.e. ‘1’, ‘2’ etc.).

### Implementation

Routines for scaffold, analog, and MCS identification, R-group decomposition, and indexing of substitution sites are implemented in Java using the OpenEye OEChem toolkit version 2.0.2 (Open Eye Scientific Software; http://www.eyesopen.com). Therefore, this toolkit is required to execute the program. All graphical components of AnalogExplorer and AnalogExplorer2 are implemented using the open source Java package JUNG version 2.0.1 ( http://jung.sourceforge.net/). Potential inconsistencies with subsequent versions of OEChem or JUNG can be avoided by using the specified versions.

### Program use

The executable program utilizes standard SD files as input and generates complete or reduced graphs for all or individual series, depending on the user’s preference. The initial graph layout is produced by the DAGLayout algorithm of JUNG ( http://jung.sourceforge.net/) and usually interactively modified for graphical analysis. The number of compounds assigned to each node and their mean potency can be viewed by navigating the graph. R-group trees representing compound subsets are generated together with the complete or reduced graph. In each R-group tree, the substituents associated with individual nodes, compounds (leaf nodes), and corresponding potency values can also be viewed. Subsets of stereoisomers, if available, are depicted using numerical indices, as discussed. Furthermore, an output file is generated reporting compounds belonging to individual subsets.

## Exemplary applications

**Figure 3.**
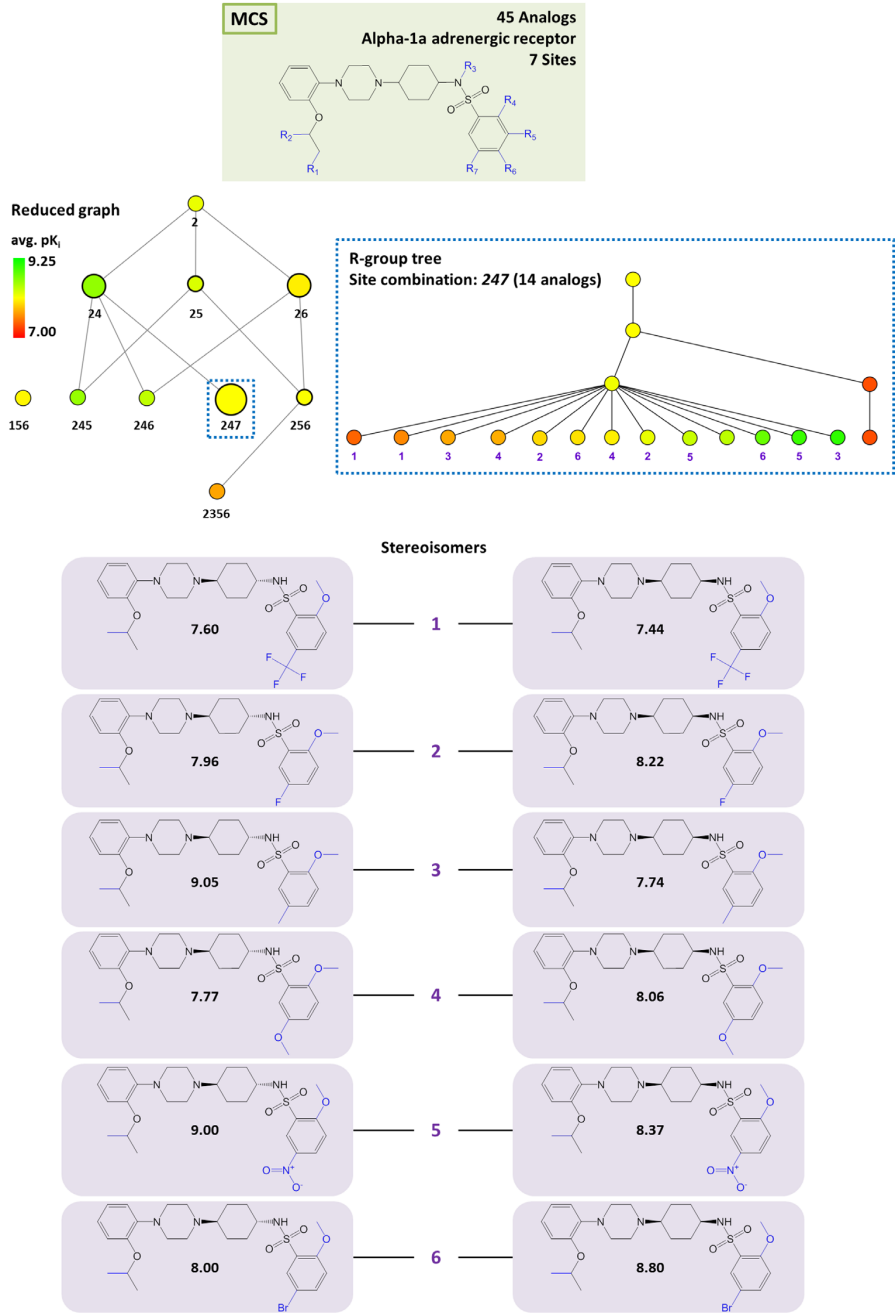
Alpha-1a adrenergic receptor ligands. At the top, the MCS for a series of 45 analogs active against alpha-1a adrenergic receptor is shown. In addition, the corresponding reduced graph (middle, left) and R-group tree for substitution site combination *247* (middle, right) are displayed. At the bottom, six pairs of stereoisomers are shown. For each compound, its pK _i_ value is given.

**Figure 4.**
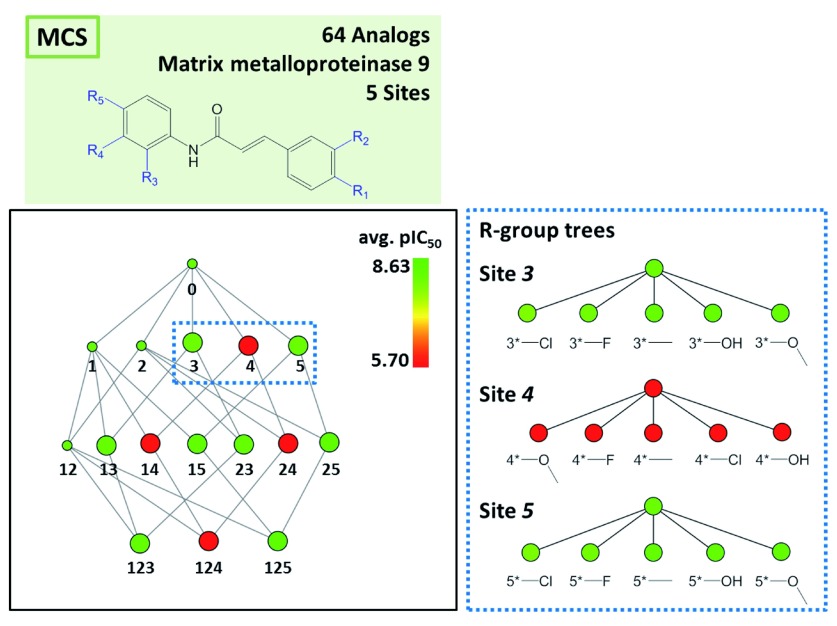
Matrix metalloproteinase 9 inhibitors. Shown is the reduced graph for a series of 64 analogous inhibitors of matrix metalloproteinase 9. R-group trees of three substitution sites (nodes *3*, *4* and *5*; dashed box) are shown on the right. For each R-group tree, substituents are provided.

**Figure 5.**
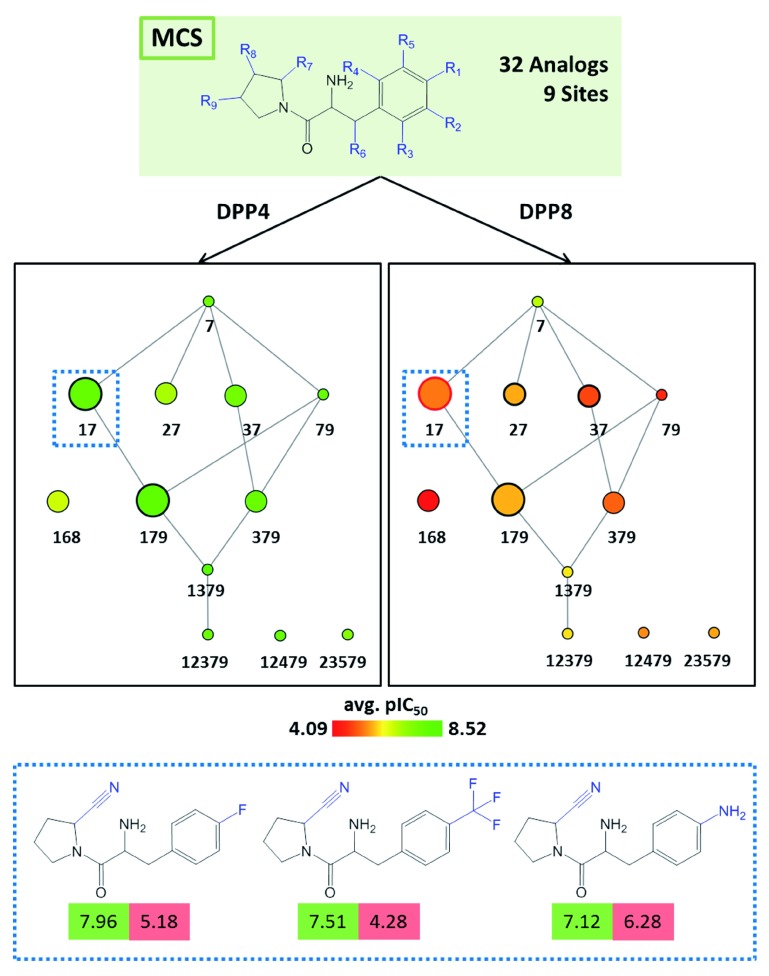
Analogs with multi-target activities. Shown are reduced graphs for analog series with inhibitory activity against dipeptidyl peptidase IV (DPP4; left) and VIII (DPP8; right). Three representative compounds associated with node *17* (dashed box) are shown at the bottom. For each compound, the potency value (pIC _50_) for DPP4 and DPP8 is reported in a green and red box, respectively.

**Figure 6.**
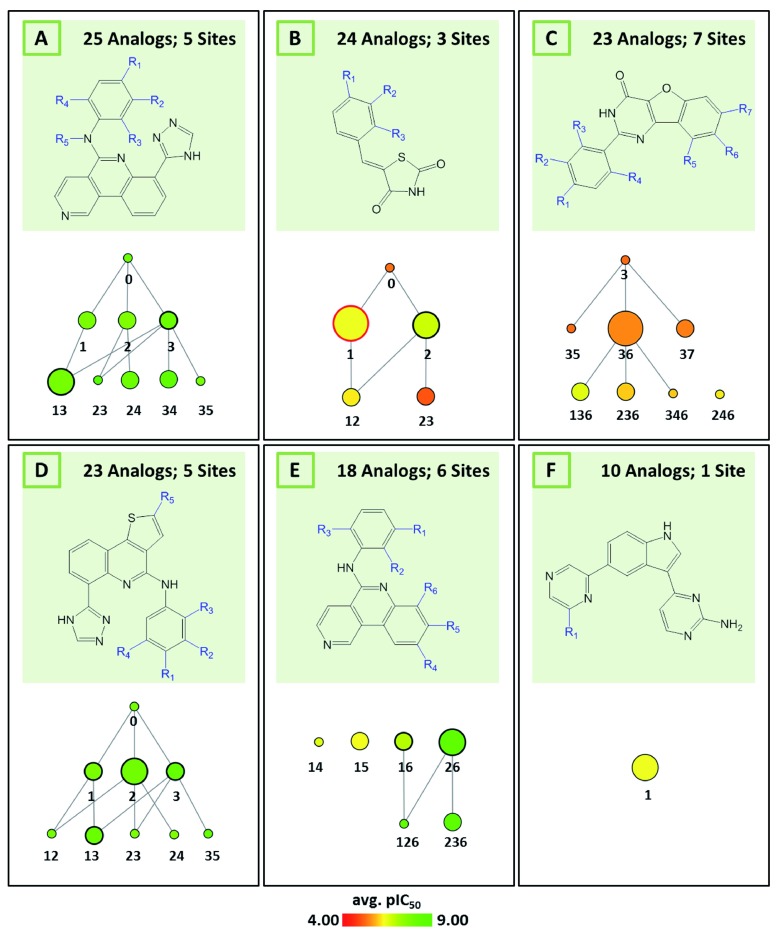
Multiple series of serine/threonine-protein kinase PIM2 inhibitors. Shown are reduced graphs for six different series of PIM2 kinase inhibitors (capital letters **A**– **F** represent series identifiers). For each series, corresponding MCS and reduced graph are shown.

**Figure 7.**
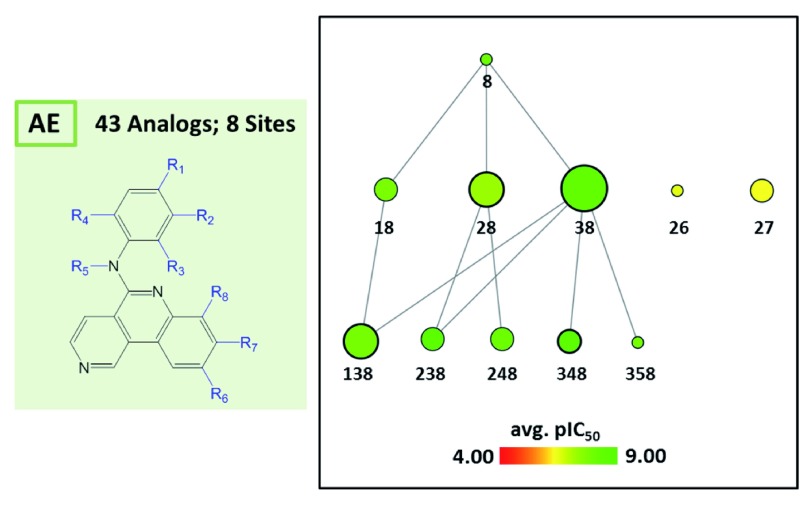
Combined series. Series A and E from Figure 6 are combined into a single series by determining their MCS yielding eight substitution sites (left). The reduced graph of the combined series is shown (right) that consists of 43 analogs.

AnalogExplorer2 can be used for different types of SAR analysis, as illustrated by a few exemplary applications. Compound data were taken from ChEMBL ^13^ version 20. Figure 3 displays the reduced graph for a series of 45 alpha-1a adrenergic receptor ligands with a total of seven substitution sites and the R-group tree for an exemplary three-site combination. The tree reveals a clear SAR pattern (with increasing potency of analogs from the bottom left to the right) and identifies six (uniquely indexed) pairs of stereoisomers among these analogs. Figure 4 provides a corresponding representation for a series of 64 matrix metalloproteinase 9 inhibitors and compares R-group trees for three substitution sites. It becomes apparent that substituents attached to R4 alone or in combination with other sites consistently yield compounds having only low potency. In Figure 5, target-specific reduced graphs are compared for a series of 32 analogs with a total of nine substitution sites and activity against two dipeptidyl peptidases (DPP4 and DPP8). The graphs generated for the same analog series display different compound potency distributions, reflecting a selectivity tendency for DPP4 over DPP8. Figure 6 shows reduced graphs for six different analog series with activity against the same kinase. The graphs reveal different structural content and a different degree of chemical exploration among these series as well as differences in the SAR information they provide. In Figure 7, two of these series are combined into a new single series by re-calculating the MCS that comprises 43 analogs with a total of eight substitution sites. The reduced graph captures the structural organization and activity information of this combined series.

## Software and data availability

The following tools and data sets are made publicly available without restrictions via a deposition on the ZENODO open access platform ^12^. Three executable files of the original AnalogExplorer program are provided for different applications including the analysis of multiple analog series from a given compound set, analysis of an individual series, and selectivity analysis (according to Figure 5). With the exception of the OpenEye OEChem library, jar files of the required external libraries are also provided. In addition, all compound sets analyzed in the original publication ^9^ are deposited. These compound sets were taken from ChEMBL version 18. Furthermore, three executable files are made available for AnalogExplorer2 (for multiple analog series, individual series, and selectivity analysis) as well as the compound sets for which graph representations are reported herein. These compounds were taken from ChEMBL version 20. A “readme” document with detailed explanations is also provided as a part of the deposition.

## Conclusions

The AnalogExplorer method was designed for the systematic organization and graphical analysis of large series of analogs, which frequently originate from lead optimization efforts. Herein, an extension of the methodology has been introduced. AnalogExplorer2 explicitly accounts for all stereoisomers during graphical analysis and SAR exploration. The AnalogExplorer2 program is made freely available to the scientific community.

## References

[1] Agrafiotis DK Shemanarev M Connolly PJ: SAR maps: a new SAR visualization technique for medicinal chemists. *J Med Chem.* 2007;50(24):5926–5937. 10.1021/jm070845m 17958407

[2] Kolpak J Connolly PJ Lobanov VS: Enhanced SAR maps: expanding the data rendering capabilities of a popular medicinal chemistry tool. *J Chem Inf Model.* 2009;49(10):2221–2230. 10.1021/ci900264n 19791782

[3] Peltason L Weskamp N Teckentrup A: Exploration of structure-activity relationship determinants in analogue series. *J Med Chem.* 2009;52(10):3212–3224. 10.1021/jm900107b 19397320

[4] Wawer M Bajorath J: Similarity-potency trees: a method to search for SAR information in compound data sets and derive SAR rules. *J Chem Inf Model.* 2010;50(8):1395–1409. 10.1021/ci100197b 20726598

[5] Agrafiotis DK Wiener JJ Skalkin A: Single R-Group Polymorphisms (SRPs) and R-cliffs: an intuitive framework for analyzing and visualizing activity cliffs in a single analog series. *J Chem Inf Model.* 2011;51(5):1122–1131. 10.1021/ci200054u 21504183

[6] Wassermann AM Bajorath J: Directed R-group combination graph: a methodology to uncover structure-activity relationship patterns in a series of analogues. *J Med Chem.* 2012;55(3):1215–1226. 10.1021/jm201362h 22248436

[7] Wassermann AM Haebel P Weskamp N: SAR matrices: automated extraction of information-rich SAR tables from large compound data sets. *J Chem Inf Model.* 2012;52(7):1769–1776. 10.1021/ci300206e 22657271

[8] Gupta-Ostermann D Bajorath J: The ‘SAR matrix’ method and its extensions for applications in medicinal chemistry and chemogenomics [v2; ref status: indexed, http://f1000r.es/3rg]. *F1000Res.* 2014;3:113. 10.12688/f1000research.4185.2 25383183 PMC4215758

[9] Zhang B Hu Y Bajorath J: AnalogExplorer: a new method for graphical analysis of analog series and associated structure-activity relationship information. *J Med Chem.* 2014;57(21):9184–9194. 10.1021/jm501391g 25333505

[10] Kenny PW Sadowski J: Structure Modification in Chemical Databases. In *Chemoinformatics in Drug Discovery* Oprea TI, (Ed.), Wiley-VCH, Weinheim, Germany,2005;271–285. 10.1002/3527603743.ch11

[11] Bemis GW Murcko MA: The properties of known drugs. 1. Molecular frameworks. *J Med Chem.* 1996;39(15):2887–2893. 10.1021/jm9602928 8709122

[12] Hu Y Zhang B Vogt M: AnalogExplorer and AnalogExplorer2. *ZENODO.* 2015 Data Source 10.12688/f1000research.7146.1 PMC4743145 26913194

[13] Bento AP Gaulton A Hersey A: The ChEMBL bioactivity database: an update. *Nucleic Acids Res.* 2014;42(Database issue):D1083–D1090. 10.1093/nar/gkt1031 24214965 PMC3965067

