# Supplementary material for: AnalogExplorer2 – Stereochemistry sensitive graphical analysis of large analog series
Source: F1000Res. 2015 Oct 9;4:Chem Inf Sci-1031. [Version 1] doi: 10.12688/f1000research.7146.1 (PMC4743145; doi:10.12688/f1000research.7146.1)
Supplement: Peer review history

# Peer review: Referee response for version 1

**DOI:** 10.5256/f1000research.7699.r11071

**Published:** 2015-11-04

**Authors:** Carleton Sage

**Affiliations:** 1 Arena Pharmaceuticals Inc, San Diego, CA, USA

The authors provide an extension to AnalogExplorer incorporating stereochemistry, and important extension of the method. This method of analysing complicated compound data sets is sophisticated and easy to use.   The approach to communicating results is intuitive both from the perspective of the trends in the experimental data with respect to the compounds that have been made, but also, in complete graph view, provide insight into important regions of the dataset that have not yet been investigated. This tool will be very useful in the exploration of medicinal chemistry datasets in both industry and academia.

                                                                      

I have downloaded and run the software on a windows workstation using both the provided datasets and internally generated ones – the software works as described with two minor issues cropped up. First, the software depends not only on the OEChem Toolkit, but also requires OEDepict (both licenses are available from Openeye Scientific). Second, the software depends specifically on the precise OEChem Toolkit java jar file (oejava-2014.Oct.2-Windows-x64.jar) – it will fail without the correct jar file.

On balance AnalogExplorer2 is a very useful tool for exploring compound data sets. It could be made more widely useful if it were to be adapted to run on an open source chemical toolkit such as RDKit. Nonetheless, I commend the authors for making their software available to the research community.

I have read this submission. I believe that I have an appropriate level of expertise to confirm that it is of an acceptable scientific standard.

# Peer review: Referee response for version 1

**DOI:** 10.5256/f1000research.7699.r10815

**Published:** 2015-10-15

**Authors:** Peter Ertl

**Affiliations:** 1 Novartis Institutes for Biomedical Research, Basel, Switzerland

Analysis of diverse molecular datasets is one of the most common tasks a computational chemist in pharmaceutical industry, but also in academia is facing daily. The tool presented in this manuscript – the AnalogExplorer2 supports this process and makes it easier. Enhancement to previously published AnalogExplorer presented here, namely support for handling of stereoisomers is indeed important, since in large number of complex SAR datasets molecules with the same constitution but different stereochemistry are present. And in some cases differences in bioactivity for different stereoisomers are significant.

Several examples shown in the manuscript illustrate nicely the powerful visualization capabilities of this tool, including maximum common substructure visualization, substituent tree visualization and selectivity analysis. These visualizations allow to understand the SAR even for the quite complex datasets.

The authors made the executable version of the tool freely available for download. This is a good news.  But since the tool is based on the OEChem library only users with a valid OEChem license would be able to use it. My recommendation for the future versions of AnalogExplorer would be to make the interface to its cheminformatics processing modular, so it would be possible to plug the tool also to open source cheminformatics toolkits like RDKit or CDK and make in this way the AnalogExplorer a truly free tool for graphical analysis of complex molecular datasets.

I have read this submission. I believe that I have an appropriate level of expertise to confirm that it is of an acceptable scientific standard.
